# Palliative care physicians’ motivations for models of practicing in the community: A qualitative descriptive study

**DOI:** 10.1177/02692163211055022

**Published:** 2021-12-17

**Authors:** Abby Maybee, Samantha Winemaker, Michelle Howard, Hsien Seow, Alexandra Farag, Hun-Je Park, Denise Marshall, Jose Pereira

**Affiliations:** 1 Department of Family Medicine, McMaster University, Hamilton, ON, Canada; 2 Division of Palliative Care, McMaster University, Hamilton, ON, Canada; 3 Department of Oncology, McMaster University, Hamilton, ON, Canada

**Keywords:** Delivery of health care, palliative care, primary health care, qualitative research, motivation, practice patterns

## Abstract

**Background:**

Internationally, both primary care providers and palliative care specialists are required to address palliative care needs of our communities. Clarity on the roles of primary and specialist-level palliative care providers is needed in order to improve access to care. This study examines how community-based palliative care physicians apply their roles as palliative care specialists, what motivates them, and the impact that has on how they practice.

**Design:**

A qualitative descriptive study using semi-structured virtual interviews of community-based palliative care specialists. We asked participants to describe their care processes and the factors that influence how they work.

**Setting/participants:**

A qualitative descriptive study using semi-structured virtual interviews of community-based palliative care physicians in Ontario, Canada was undertaken between March and June 2020. At interview end, participants indicated whether their practice approaches aligned with one or more models depicted in a conceptual framework that includes consultation (specialist provides recommendations to the family physician) and takeover (palliative care physician takes over all care responsibility from the family physician) models.

**Results:**

Of the 14 participants, 4 worked in a consultation model, 8 in a takeover model, and 2 were transitioning to a consultation model. Different motivators were found for the two practice models. In the takeover model, palliative care physicians were primarily motivated by their relationships with patients. In the consultation model, palliative care physicians were primarily motivated by their relationships with primary care. These differing motivations corresponded to differences in the day-to-day processes and outcomes of care.

**Conclusions:**

The physician’s personal or internal motivators were drivers in their practice style of takeover versus consultative palliative care models. Awareness of these motivations can aid our understanding of current models of care and help inform strategies to enhance consultative palliative care models.

**What is already known about the topic?**

- Previous research has described models of palliative care delivery on a spectrum, with varying levels of family physician (generalist level) and palliative care (specialist level) physician involvement.
- The models vary from a consultation model, where the palliative care physician provides mentorship and recommendations to the family physician, to a takeover model, where the palliative care physician “takes over” the patient’s care from the family physician.
- Factors influencing the models in which palliative care physicians work are unclear.

**What this paper adds?**

- Physicians’ personal satisfaction to either provide direct patient care or support family physicians with specialized knowledge appears to drive their motivation toward a practice model. Day-to-day activities followed from these motivations and were different between models.

**Implications for practice, theory, or policy**

- The findings suggest that system-level strategies aiming to change the processes and outcomes of palliative care need to consider underlying motivators and philosophies of physician practice.
- Appreciating physicians’ motivations allows for an understanding of current models of care and informs strategies to enhance consultative palliative care models in the community.

## Introduction

There is growing recognition internationally that palliative care services need to be provided by both specialist- and generalist-level providers (i.e. primary care providers).^1–4^ The demand for palliative care is growing due to the aging population and as it is applied earlier in the illness trajectory across cancer and non-cancer diagnoses.^5–8^ Specialist-level palliative care requires advanced training and experience, often delivered by interprofessional teams, to meet the needs of patients and families with complex needs.^9^

However, not all patients present with complex needs and most may have their needs met by generalist providers.^10^ This non-specialist palliative care is referred to by different terms, including the palliative care approach, primary palliative care, and generalist palliative care.^4,11^ Generalist palliative care can be effectively provided by health care professionals, such as primary care providers or home care nurses,^12^ provided they have core competencies in the area and are supported by specialist palliative care teams.^11,13,14^ Coordination is needed between these two levels to best meet patients’ and families’ needs.^15,16^

Pereira *et al.* proposed a framework to characterize different palliative care provider models.^17–19^ The framework represents a continuum with a consultation model and a takeover model at either ends of the spectrum. In the consultation model, the palliative care clinician (e.g. physician, nurse, or multidisciplinary team), provides clinical support and coaching to the patient’s Most Responsible Physician. They make recommendations and provide advice to the Most Responsible Physician as required until needs are addressed. In a takeover model, the palliative care clinician replaces the original Most Responsible Physician, who is now no longer or only peripherally involved in the care. Brown *et al.* validated that the models are applicable to physicians using administrative data in Ontario, Canada.^20^ The differing physician models have also been shown in other Canadian survey research^21^ and in other settings and countries.^22,23^

With respect to community-based palliative care, to date, there is little understanding of why palliative care physicians work in a consultation versus a takeover model. Furthermore, there is a paucity of information that describes the way palliative care physicians operationalize these different practice models in their daily clinical work.

## Methods

### Research question

Our study aimed to describe the day-to-day care processes of community-based palliative care physicians working in different practice models and to better understand their underlying motivators of why they work the way they do.

### Study design

We conducted a qualitative descriptive study using semi-structured interviews because we sought to understand the complex process of why and how one works from the perspective of the physicians involved.^24^

### Setting and participants

Study participants were palliative care physicians who were working or had previously worked in a community setting, specifically providing home visits, in the province of Ontario, Canada. To be eligible, physicians were required to be certified by the College of Family Physicians of Canada with either a year of added competence or certificate of added competence in palliative care (CAC-PC).

### Recruitment/sampling

The clinician researchers initially identified potential participants based on their own networks and knowledge of different practice models that colleagues across the province used. This initial group of potential participants were invited to participate in the study (purposive sampling) and then asked to identify other palliative care physicians who practiced in similar models (snowball sampling). Recruitment stopped by the 14th interview, as new emerging themes did not develop from interviews thereafter. An “information power” approach was applied, defined as 12–18 participants with similar profiles and experiences discussing a narrow question.^25^

### Data collection

The discussion guide was created through several discussions and iterations by the research team. As a taxonomy to guide the inquiry of the participant’s care processes, we used the framework by Pereira *et al.*^17^ which describes issues such as prescribing and after-hours coverage in different models.^20^ The discussion guide included questions to elicit a description of day-to-day processes and operations of the participant’s clinical work, followed by questions regarding the influencing factors of the participant’s self-described practice. We introduced the concept of practice model at the end of the interview, where the participant was asked to view a diagram of the spectrum and identify their model of care.

Each palliative care physician participated in a 60-min semi-structured virtual interview via ZOOM between March and June 2020 and was digitally recorded. Interview notes were created and the recordings were reviewed afterwards to supplement the notes.

### Data analysis

We used a theoretical thematic analysis approach since the themes were researcher-driven by the interview guide which was intentionally structured from a framework.^26^ Data were analyzed in relation to the participants’ description of how they work and the factors that influenced how they work. Two researchers (SW, AM) independently coded each set of interview notes and identified emerging themes. The two researchers then agreed on emerging themes by consensus. The audio recordings and notes were reviewed by two additional researchers (AF, HJP) for further verification of the coding decisions. The emerging themes were then discussed with all the authors for further reflection and interpretation. Salient quotes were selected from each interview to demonstrate themes. No qualitative analytic software program was used.

### Ethical issues

Ethics approval for this study was obtained from the Hamilton Integrated Research Ethics Board in Hamilton, Ontario, Canada on December 18, 2019 (#8118). We considered the potential issues of being negatively perceived to speak about monetary incentives, different practice models in the system, or other providers in the community, which is why data reporting kept responses anonymous.

## Results

### Participant characteristics

Fourteen palliative care physicians were interviewed. Most (64%; 9/14) of the interviewees were in their first 10 years of practice and 71% (10/14) worked in multiple care settings (e.g. community hospice and in-patient hospital). Three participants had a combined practice which included family medicine. Eight participants worked in an urban setting (defined as population > 130,000 people). Half of the participants were remunerated by a fee-for-service (FFS) payment model, and half were salaried (Tables 1 and 2).

**Table 1.** Characteristics of the 14 interview participants’ palliative care practice.

Self-identified model	Stage of practice^a^	Rural vs urban (population >130,000)	Multiple care settings^b^	Combined practice with family medicine	Funding model
Consultation	Early	Urban	Yes	Yes	Salary
Consultation	Late	Urban	Yes	No	Salary
Consultation	Late	Urban	Yes	No	Fee-for service
Consultation	Late	Both	No	No	Salary
Takeover	Early	Urban	Yes	No	Fee-for service
Takeover	Early	Urban	Yes	Yes	Fee-for service
Takeover	Late	Urban	No	No	Salary
Takeover	Late	Both	Yes	No	Salary
Takeover	Early	Urban	Yes	No	Fee-for service
Takeover	Early	Rural	Yes	Yes	Fee-for service
Takeover	Early	Rural	Yes	No	Fee-for service
Takeover	Early	Urban	Yes	No	Fee-for service
Transition	Early	Both	No	No	Salary
Transition	Early	Both	No	No	Salary

a “Early” deemed less than 10 years in practice, “Late” deemed as greater than 10 years in practice.

b Multiple care settings (e.g. residential hospice, hospital).

**Table 2.** Aggregate table of participant characteristics.

	Consult (*n* = 4)	Takeover (*n* = 8)	Transition (*n* = 2)
Stage of practice^a^			
Early	1	6	2
Late	3	2	0
Community size			
Rural	0	2	0
Urban (>130,000)	4	5	0
Both	1	1	2
Multiple care settings^b^			
Yes	3	7	0
No	1	1	2
Combined practice with family medicine			
Yes	1	2	0
No	3	6	2
Funding model			
Salary	3	2	0
Fee-for-service	1	6	2

a “Early” deemed less than 10 years in practice, “Late” deemed as greater than 10 years in practice.

b Multiple care settings (e.g. residential hospice, hospital).

Four participants identified themselves on the framework as working in a consultation model, eight identified as working in a takeover model and two identified as transitioning from a takeover model to a specialist model. Generally, the participants described working in one care model and did not describe working flexibly across the continuum of care models. None of the participants described varying their work across the care model spectrum according to patient or provider needs.

### Major themes

Participants described their day-to-day care processes in a way that was deeply intertwined with their personal philosophy of their role as palliative care providers. The analysis revealed three major themes, or differences, between palliative care specialists who work in a takeover model and a consultation model; fundamental differences in their philosophical drivers ultimately lead to differences in both processes and outcomes of care.

#### Theme 1: Differences in philosophical drivers

The main difference between palliative care physicians working in a consultation model versus a takeover model was their underlying motivators, or philosophy of palliative care provision (Table 3).

**Table 3.** Summary of themes that emerged from interviews by palliative care practice model.

	Consultation	Takeover
**Philosophical drivers**		
Focus of relationship	Relationships with primary care	Relationships with patients
Role of family physicians and home care teams	Integral, non-negotiable	Family physicians unavailable, absent
**Processes**		
Communication with family physician	Required	Limited
Availability of palliative care physician	Second-line after family physician	First-line, always available
Collaboration	Multidisciplinary team	Solo provider
**Outcomes**		
Continuity of care for patient	Provided by family physician	Provided by palliative care physician
Challenges	Navigating supportive role	Demand
Systems advocacy	Capacity building in primary care	Training more, specialized palliative care physicians

##### Sub-theme: Focus of relationship

Relationships with others were central to how participants described their motivation. Palliative care physicians in the takeover model were primarily motivated by their own relationships with patients. The most rewarding aspect of their work was helping patients navigate their journey toward end-of-life. These physicians felt their role was integral to providing a good “final chapter” of each patient’s life.

*“Being able to be with them through this journey and getting to know them and their families is a rewarding and unique thing.” (#4)*

*“Patients only have one shot at good end-of-life care and I enjoy writing a good ending and final chapters of each patient’s life.” (#9)*

In contrast, palliative care physicians in a consultation type model were primarily motivated by their own relationships with primary care providers. They described satisfaction from connecting with their primary care colleagues and assisting them in providing palliative care for their patients. They saw themselves as mentors for primary care with the goal of capacity building.

*“It’s not just about the care of the patient, it’s about learning where the family doctor needs help, so ideally they can do it next time.” (#3)*

*“I get satisfaction from connecting with my family medicine colleagues.” (#1)*

##### Sub-theme: Role of family physicians and home care teams

The role of the family physician in providing palliative care was also a fundamental philosophical difference between the two groups. In the consultation model, which supports primary level palliative care provided by primary care providers, the palliative care physician promoted the involvement of the family physician in their patient’s care. The family physician’s role was thought to be integral and non-negotiable. These palliative care physicians identified benefits of their primary care colleagues providing palliative care and felt that they could manage even some of the most difficult palliative care cases, with specialist support.

*“I have high expectations of my primary care colleagues and believe that they can do this [palliative care] with our support.” (#3)*

*“It is a theoretical requirement of the program that they all [family physicians] do home visits.” (#7)*

In the takeover model, many interviewees felt that family physicians did not want to be involved with palliative care, were not able to provide their patients with after-hours support and did not provide home visits. They spoke of an overall paucity of primary care engagement and involvement. Some interviewees even described “giving up” on working directly with family physicians.

*“There is an absence of family doctors doing house calls and providing after-hours care.” (#9)*

*“If we rely on the family physician, we have more variability in the level of care that patients receive, and I don’t feel comfortable with that.” (#8)*

#### Theme 2: Difference in processes

Through the description of care processes, or day-to-day work, differences between models were noted in communication with family physicians, availability of the palliative care physician, and collaboration with others in the care team.

##### Sub-theme: Communication

In the consultation model, palliative care physicians purposefully engaged the family physician to negotiate a working relationship either at the time of referral or after the first consultation. In the takeover model, there was limited communication with the family physician as the palliative care physician took over care immediately after their consultation. Many interviewees in this model felt that time was wasted on engaging primary care and navigating a shared role in patient care. An interviewee working in a takeover model described the difficulty of having two providers of palliative care: “*If the patient is closer to end of life, there needs to be only one hand on the steering wheel*” *(#9).*

##### Sub-theme: Availability

Palliative care physicians in the consultation model described that they generally complete home visits to patients only as needed and requested by the family physician. They positioned their role as a “second line” for family physicians and community nurses. They were not directly available to patients and their families. In the takeover model, physicians, or their group service, were available “24/7” and patients and their families had direct access to them. This was cited as a source of burnout for many of the interviewees who practiced in a takeover model.

##### Sub-theme: Collaboration

Palliative care physicians in the consultation model described working as part of a team, which included formal home-care services such as visiting palliative care nurses and family physicians. There was intention to liaise with these team members frequently, resulting in a team approach to care. In the takeover model, palliative care physicians often completed consults alone and engaged minimally with family physicians and other members of the care team.

#### Theme 3: Difference in outcomes

Differences between models were noted in the resulting outcomes of working in different ways, with respect to continuity of patient care, challenges of working in the model, and implications for system advocacy.

##### Sub-theme: Continuity of care

In both models, patients received enduring continuity, although the source of this continuity differed. In a consultation model, the family physician was positioned as the patient’s main source of continuity. In the takeover model, the palliative care physician provided continuity, with a rhythm and cadence of follow-up care that was more frequent and regular. Interviewees in the takeover model derived deep satisfaction from this role—“*I feel like I’m the patient’s constant*” *(#4).*

##### Sub-theme: Challenges

Participants described different challenges in the two different models of care. In the takeover model, the palliative care physicians positioned themselves as the main provider and experienced challenges around demand. They described being “always available” and alluded to the possibility of burnout. In the consultation model, the main challenge was negotiating and navigating their supportive role with primary care. One palliative care physician described this as the “counter-cultural” model, a constant struggle to resist the pressure to take over care completely from the family physician. They also described feeling like they had less control over patient care as a result of deliberately standing back and allowing the family physician to manage palliative care issues. Palliative care physicians in a consultation model also described the lack of emotional reinforcement*—*“*if I do my model well, I have a very minimal emotional role with these patients and their family . . . palliative care doctors find that difficult*” (#7). Sources of job satisfaction and dissatisfaction were also connected to both their underlying philosophical beliefs and a result of the day-to-day work processes. Interviewees who worked in a care model that misaligned with their core philosophy, described dissonance with their work processes, and a significant degree of personal distress.

##### Sub-theme: Systems advocacy

Palliative care physicians in both models were concerned with equity and accessibility of palliative care but proposed different solutions. In the consultation model, palliative care physicians felt they were a finite resource and the focus of their advocacy was capacity building of their family medicine colleagues—“*I’ll know I’ve done a good job when I am out of a job – when family physicians no longer need my advice*” (*#3*). In the takeover model, palliative care physicians felt there is a need to provide more direct patient care and that the focus should be on increasing patient access to palliative care physicians by training more specialists.

## Discussion

### Main findings

In this qualitative study examining provider motivations for model of practice in the community, we found palliative care physicians have varying underlying personal motivators that influence their practice model, which are associated with different challenges and rewards. In the takeover model, palliative care physicians were primarily motivated by their relationships with patients. In the consultation model, palliative care physicians were primarily motivated by their relationships with primary care, specifically with respect to mentorship and capacity building. The different practice model also influenced the day-to-day processes and overall outcomes of their care, including differences in sources of job satisfaction, challenges, and systems advocacy.

### Implications

Our findings about the role of personal motivations in influencing the practice of palliative care physicians have implications for health care system planners. It is noteworthy that palliative care physicians who worked in the takeover model perceived that family physicians preferred not to be involved in palliative care. If health systems aspire to encourage the development of generalist palliative care, they will need to expose medical trainees to such models during undergraduate and postgraduate training.^27^ Participants who described taking over care from family physicians also recognized a current lack of confidence to provide palliative care amongst family physicians. A study of primary care physicians in 10 countries found that over half indicated that they felt uncomfortable providing palliative care.^28^ To address this, regulatory bodies will need to advocate for more salaried positions for palliative care specialists that include explicit deliverables for primary care mentorship and capacity building, not just for direct patient care.

Given an aging population and the benefit for early palliative care provision, there is a strong need for robust primary-level palliative care capacity provided by health care professionals across care settings, professions and specialty areas, and supported by specialist palliative care teams.^1,5,6,8,13,16^ An ethical imperative for this has also been highlighted.^29^ Previous research has examined the barriers and enablers of family physicians providing primary palliative care^21,30–32^ and different models of palliative care provision.^1,20,33^ Our study found that personal motivations of physicians influences their practice model and contributes to the literature of why palliative care physicians work the way they do and why the various models of care exist. Appreciating these motivations allows deeper understanding of current models of care and informs strategies to support primary-level care.

### Limitations

This study is confined to one jurisdiction in Canada and does not reflect other Canadian or international jurisdictions. However, we believe that the findings and lessons from the study are pertinent for health system planners and palliative care service providers internationally. This study only interviewed palliative care physicians, but other interprofessional health care professionals also play a key role in palliative care delivery and their perspectives on the different models of care were not included; this ought to be explored in future research. There may be other potential factors that influence the practice model that the study may not have been able to uncover, including availability of community resources and financial or remuneration aspects.

## Conclusion

Personal motivations of community-based palliative care physicians influences their involvement in takeover versus consultation models. Physicians derived job satisfaction from either direct patient care or from leveraging specialized knowledge to enable generalists. Day-to-day activities followed from these motivations and were different between models. Each model was associated with personal and practical challenges. The findings suggest that system-level strategies aiming to change the processes and outcomes of palliative care need to consider underlying personal motivators and philosophies of physician practice.
